# Repeatability, Reproducibility, and Comparability of Subjective and Objective Measurements of Intraocular Forward Scattering in Healthy Subjects

**DOI:** 10.1155/2015/925217

**Published:** 2015-06-14

**Authors:** Ayaka Iijima, Kimiya Shimizu, Hidenaga Kobashi, Aya Saito, Kazutaka Kamiya

**Affiliations:** Department of Ophthalmology, University of Kitasato School of Medicine, Kanagawa 252-0374, Japan

## Abstract

*Purpose*. To assess the repeatability, reproducibility, and comparability of measurements of subjective and objective forward scattering in healthy subjects.* Methods*. We prospectively examined twenty eyes of 20 healthy volunteers (7 men and 13 women; ages, 28.4 ± 4.1 years). The logarithmic straylight value (log(*s*)) and the objective scattering index (OSI) were measured with a straylight meter (C-Quant) and a point-spread function meter (OQAS), respectively.* Results*. The 95% limits of agreement (LoA) between first and second measurements ranged from −0.211 to 0.207 for the C-Quant and from −0.302 to 0.477 for the OQAS. The intraclass correlation coefficients for the repeatability of the log(*s*) and OSI measurements were 0.815 and 0.926, respectively. The mean difference between examiners was −0.051 ± 0.133 (95% LoA; −0.311 to 0.209) for the C-Quant and 0.080 ± 0.307 (−0.522 to 0.682) for the OQAS. There was a modest, but significant, correlation between the log(*s*) and the OSI (Spearman correlation coefficient *r* = 0.498, *p* = 0.026).* Conclusions*. The C-Quant and the OQAS provide good repeatability and reproducibility, although the OQAS measurement provides a slightly higher ICC than the C-Quant measurement. The subjective forward scattering may be to some extent expressed in the objective forward scattering in healthy subjects.

## 1. Introduction

Optical aberrations and light scattering can lead to image quality degradation on the retina, resulting in the deterioration of visual performance, even in younger healthy eyes, because the optical media of the whole eye are not complete. Optical aberrations were well recognized by the Hartmann-Shack aberrometer, but the scattering of light has not so far been fully understood. Since the forward light scattering is considered to have a more direct effect on visual performance than the backward light scattering, this measurement could provide valuable insights into visual function. Accordingly, it is of importance to quantify this forward scattering in a clinical setting. Currently, two instruments are commercially available to quantify the forward light scattering; one is the straylight meter (C-Quant, Oculus, Optikgeräte, GmbH, Wetzlar, Germany) which psychometrically assesses the intraocular forward scattering based on the compensation method [1], and the other is the point-spread function (PSF) meter (Optical Quality Analysis System (OQAS), Visiometrics, Terrassa, Spain), which assesses it objectively by means of the double-pass method [2]. However, the relationship between the subjective and objective intraocular forward scattering has not so far been elucidated in healthy subjects. Although both the methodology and the units of the two measurements for intraocular forward scattering were different, it is meaningful to know how the different measurement techniques correlate with each other in clinical use. Moreover, accurate and precise measurements of intraocular scatter are mandatory for the analysis of detailed visual performance. The purpose of the current study is to prospectively assess the repeatability, reproducibility, and comparability of subjective and objective intraocular forward scattering measured by the straylight meter and the PSF meter in an ophthalmologically normal population.

## 2. Materials and Methods

Twenty eyes of 20 healthy volunteers, who were doctors, nurses, or clerical staff working at Kitasato University Hospital, were examined in this prospective study. No subject had a history of ocular surgery, trauma, or disease except for myopia, hyperopia, and/or astigmatic ametropia. Exclusion criteria were as follows: a history of prior intraocular and corneal surgery and trauma, slit-lamp microscopy showing positive evidence of corneal disease that could affect the outcome, or contact lens wear. Random selection of only one eye per subject was conducted for statistical analysis. All participants were Asians with brown irises, who had a corrected visual acuity of 20/20 or more. A sample size of 20 subjects with 3 observations per subject achieves 83% power to detect an intraclass correlation of 0.90 under the alternative hypothesis when the intraclass correlation under the null hypothesis is 0.75 using an *F*-test with a significance level of 0.05. The study was approved by the Institutional Review Board of Kitasato University and followed the tenets of the Declaration of Helsinki. Written informed consent was obtained from all volunteers after an explanation of the purpose, risks, discomfort, and steps of the study was given.

### 2.1. Subjective Assessment of Intraocular Forward Scattering

The retinal straylight was measured with the C-Quant straylight meter (Oculus Optikgeräte, GmbH, Wetzlar, Germany). This device uses the compensation comparison method described by Franssen et al. [1]. This method comprises a psychometric function designed to describe the (stochastic) characteristics of the responses. It is more suitable for clinical examination than the instrument that works with the direct compensation method. The center of the test field is divided in halves. When the compensation light is presented to one-half, no compensation light is presented to the other. Outside the center is a ring-shaped flickering light source, which serves as the straylight source. When the subject is tested, one-half of the center has counter-phase flickering, and the other has not. The subjects are asked to choose which semicircle is flickering more strongly and to press the button on the left or the right side of the device. The straylight meter will change the luminance of the stimulus and counter-phase modulating light automatically until the two halves are balanced. To obtain the straylight value, this process is repeated three times with different levels of compensation light, resulting in a logarithmic straylight value, which is abbreviated as log(*s*). Only when the estimated standard deviation was lower than 0.08 and the quality factor was higher than 1.00, was the measurement accepted [3]. All log(*s*) measurements were performed without glasses or contact lenses, while the other eye was covered. Cleaned trial glasses were used if necessary.

### 2.2. Objective Assessment of Intraocular Forward Scattering

The objective scattering index (OSI) was measured with the Optical Quality Analysis System (Visiometrics, Terrassa, Spain) for a 4.0-mm pupil. The manifest refractive error of the subjects was fully corrected during these measurements, the spherical error automatically by the double-pass system, and the cylindrical error with an external lens, since the uncorrected refractive error directly affects the optical outcome of the system. The objective scattering index (OSI) is an objective evaluation of intraocular scattered light. The index is calculated by evaluating the amount of light outside the double-pass retinal intensity PSF image in relation to the amount of light on the center. In the particular case of the instrument OQAS, the central area selected was a circle of a radius of 1 minute of an arc, while the peripheral zone was a ring set between 12 and 20 minutes of an arc [4]. The OSI for normal eyes would be around 1, while values over 5 would represent highly scattered systems.

### 2.3. Assessment of Repeatability, Reproducibility, and Comparability

Evaluations of intrasession repeatability, intersession repeatability, and reproducibility were assessed by the Bland-Altman method [5]. The 95% limits of agreement (LoA) were calculated as the mean difference ± 1.96 standard deviation (SD). Three consecutive sets of measurements with the two devices in three different sessions were taken in all subjects by a single experienced examiner (Ayaka Iijima). These sessions were performed at 1-day intervals. We used the averaged values of these 9 measurements of the log(*s*) and the OSI for analysis. Additionally, three consecutive sets of measurements with the two devices in a single session were also made in these subjects by another experienced examiner (Aya Saito). The sequence of measurements with the two instruments and the order in which examiners 1 and 2 took the measurements were randomized.

Subjects were instructed to close their eyes just before each measurement. The examiner confirmed that the head was, as far as possible, held upright. The rooms were kept in semidarkness to facilitate fixation. In the assessment of repeatability, all original data for one examiner were used. For the assessment of reproducibility (between examiners), the averaged measurements for each examiner were used since this approach is used in clinical practice. The data obtained were used for comparisons among the first, second, and third measurements within the first session (intrasession repeatability) and comparisons between sessions (intersession repeatability).

The intraclass correlation coefficient (ICC) is determined on the basis of analysis of variance for mixed models for each situation as proposed by Bartko and Carpenter Jr. [6]. The ICCs range from 0 to 1 and are commonly classified as follows: ICC less than 0.75 = poor repeatability; 0.75 to 0.90 = moderate repeatability; 0.90 or more = high repeatability [7]. To assess the comparability of the two measurements, the relationship between two sets of data was analyzed by Spearman's rank correlation test. All statistical analyses were performed using SPSS (SPSS Inc., Chicago, IL, US). Sample size calculation was performed using PASS 2008 (NCSS, Utah, USA). The results are expressed as means ± SD, and a *p* value less than 0.05 was considered statistically significant.

## 3. Results

### 3.1. Study Population

The demographic data of the study population are summarized in Table 1. Of the 20 volunteers, 13 were women and 7 were men. The mean subject age was 28.4 ± 4.1 years (range, 22 to 40 years). The mean log(*s*) was 0.848 ± 0.100 (range, 0.697 to 1.030). The mean OSI was 0.899 ± 0.369 (range, 0.422 to 1.589).

### 3.2. Intrasession Repeatability

**Figure 1:**
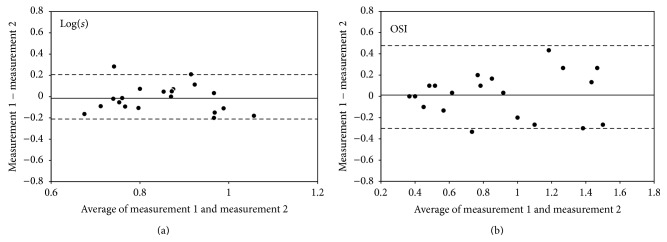
Bland-Altman plots of the repeatability of measurements of intraocular forward scattering between the first and second measurements. (a) log(*s*) obtained by straylight meter, (b) OSI obtained by point-spread function meter. The solid line indicates the mean difference. The upper and lower dotted lines represent the 95% LoA.

The ICCs for the repeatability of the log(*s*) measurements by the straylight meter and the OSI measurements by the PSF meter were 0.815 (95% LoA, 0.610 to 0.921) and 0.926 (95% LoA, 0.843 to 0.968), respectively. The mean of the differences and the corresponding 95% LoA for the log(*s*) and OSI within the first session, when the first and second measurements, the first and third measurements, and the second and third measurements were compared, are shown in Table 2. The mean difference between the first and second measurements of intraocular forward scattering was −0.002 ± 0.106 (95% LoA, −0.211 to 0.207) for the straylight meter and 0.088 ± 0.199 (95% LoA, −0.302 to 0.477) for the PSF meter (Figure 1).

### 3.3. Intersession Repeatability

The mean of the differences and the corresponding 95% LoA for the log(*s*) and OSI when the first and second sessions, the first and third sessions, and the second and third sessions were compared are shown in Table 3.

### 3.4. Reproducibility

**Figure 2:**
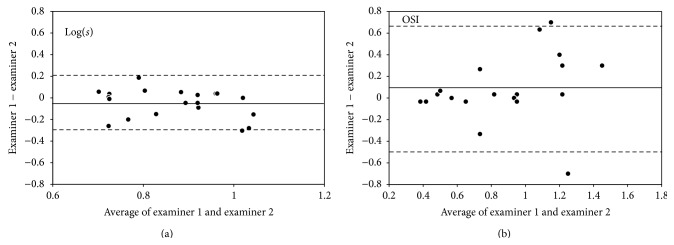
Bland-Altman plots of the reproducibility of measurements of intraocular forward scattering between examiners. (a) log(*s*) obtained by straylight meter, (b) OSI obtained by point-spread function meter. The solid line indicates the mean difference. The upper and lower dotted lines represent the 95% LoA.

The mean difference in the intraocular forward scattering between examiners was −0.051 ± 0.133 (95% LoA, −0.311 to 0.209) for the straylight meter and 0.080 ± 0.307 (95% LoA, −0.522 to 0.682) for the PSF meter (Figure 2). The between-examiner reproducibility assessments showed a trend similar to the intrasession repeatability assessments.

### 3.5. Comparability

**Figure 3:**
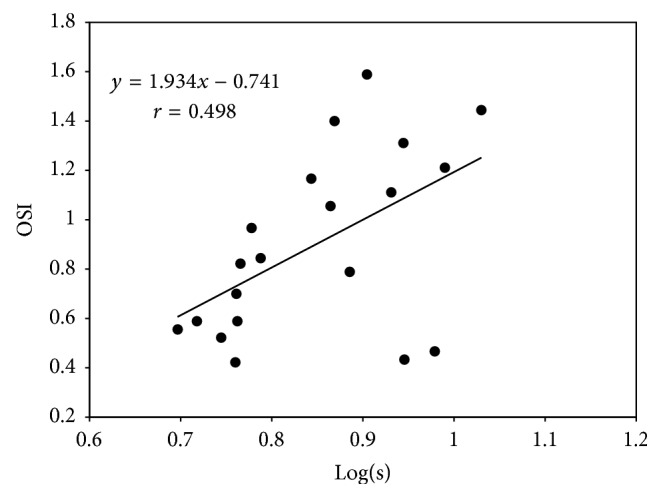
A scatter plot graph showing a modest but significant correlation between log(*s*) and OSI (Spearman correlation coefficient *r* = 0.498, *p* = 0.026).

We found a modest, but significant, positive correlation between the log(*s*) and the OSI (Spearman correlation coefficient *r* = 0.498, *p* = 0.026) (Figure 3).

## 4. Discussion

In the current study, our results support the view that both the log(*s*) obtained by the C-Quant and the OSI obtained by the OQAS showed moderate and high ICCs, respectively, suggesting that both these instruments also provide good repeatability of measurements of intraocular forward scattering for clinical use. Our present findings regarding the repeatability with the C-Quant and OQAS are in line with those of previous studies [1, 8–13]. With regard to the straylight meter, Franssen et al. [1] demonstrated that the mean log(*s*) of the repeated-measures SD was 0.07. Cerviño et al. [8] reported that the mean log(*s*) of the intrasession SD in the 10 consecutive measurements and that of the intersession SD in the 5 sessions were 0.07 and 0.05, respectively, suggesting that the C-Quant straylight meter measurements are repeatable and reliable for the assessment of the log measurements. Guber et al. [9] showed that the ICC of 5 repeated measurements was 0.83, which was comparable with our current ICC findings. With regard to the PSF meter, Saad et al. [10] showed, in a study of normal, cataractous, and postrefractive surgery eyes, that the repeatability limit (percentage of mean value) ranged between 20.9% and 56.1% for the OSI. Vilaseca et al. [11] reported that the mean coefficient of repeatability of OSI was 0.11. Kamiya et al. [12] demonstrated that the means of the intrasubject SD of the 3 consecutive measurements of OSI were 0.16. They [13] also stated, in a study of 20 normal eyes, that the mean difference between two consecutive measurements of OSI was −0.02 ± 0.17 (95% LoA, −0.35 to 0.32).

With regard to the intersession repeatability, the mean differences and the 95% LoA obtained between different sessions and within the first session are comparable, indicating that the intrasession and intersession repeatabilities of the C-Quant and the OQAS are almost the same and that the repositioning of the subject and the realignment of the eye between sessions do not introduce any additional variation into the measurements with either device.

Judged on our current findings of the ICCs, the repeatability of the OQAS measurements is slightly better than that of the C-Quant measurements. The straylight meter depends on the subjective responses of subjects, whereas the PSF meter does not. In addition, the former device requires instructions for the subjects, and measurement with it is relatively time consuming, especially when the test needs to be repeated several times. Hirnschall et al. [14] stated that the average time for one measurement with the straylight meter was 2 minutes—far longer than that with the PSF meter, even when the time required to instruct the patient on the procedure was not taken into account. We assume that the slightly better repeatability of the OQAS measurements, as evidenced by the higher ICC, results largely from the independence of subjective responses and from the shorter time required for the measurements in this study.

As far as we can ascertain, this is the first published study to quantitatively assess the relationship between the log(*s*) and the OSI, which represent subjective and objective intraocular forward scattering, respectively, in an ophthalmologically normal population. The C-Quant psychometrically measures the retinal straylight as a forward light scattering in a large visual angle of 5 to 10 degrees by the compensation comparison method. By contrast, the OQAS measures the forward light scattering by analyzing the retinal image of a point source of light obtained after focalization of an infrared beam in smaller visual angle within 20 minutes of an arc by the double-pass method. Therefore, the implications of the values measured with the straylight and PSF meters are substantially different, although both obtained values are related to the intraocular forward scattering. We found a modestly significant correlation between the values of the log(*s*) and the OSI in the present study. These findings indicate that the subjective forward scattering may be reflected by the objective forward scattering to some extent but that the subjective scattering does not necessarily coincide with the objective scattering for a clinical use.

Hirnschall et al. [14] reported, in a study of 50 eyes with posterior capsular opacification after cataract surgery, that the correlation between the straylight and PSF meters was moderate but close to the significance level. The straylight meter measures a larger visual angle (5 to 10 degrees) than the PSF meter (within 20 minutes of an arc). The discrepancy may be attributed to the differences of the visual angle (small versus large) as well as the methodology of the measurements (psychometric versus objective). It should be noted that these scattering parameters obtained by the straylight meter and the PSF meter are not interchangeable for the assessment of intraocular forward scattering.

The limitation of this study is that we did not assess the accuracy of the intraocular forward scattering measurements with the C-Quant and the OQAS. This inevitably raises the question of which method would be more valid; however, with the lack of a gold standard, we cannot provide an answer to this question at this time.

In summary, we systematically evaluated the repeatability, reproducibility, and comparability of the intraocular forward scattering obtained from the C-Quant and the OQAS in an ophthalmologically normal population. Our results support the view that both measurements of the intraocular forward scattering proved to provide good repeatability, although the OQAS measurement provides a slightly higher ICC than the C-Quant measurement, and the view that the two devices were moderately correlated in healthy subjects, suggesting that the subjective forward scattering may reflect the objective forward scattering to some extent in a clinical setting.

## Figures and Tables

**Table 1:** Demographic data of the study population.

Demographic data	
Number of subjects	20

Age (years)	28.4 ± 4.1 years (range, 22 to 40 years)

Gender (% female)	65.0%

Manifest spherical equivalent (D)	−2.13 ± 2.15 D (range, −6.5 to 0 D)

LogMAR corrected visual acuity	−0.08 ± 0.04 (range, −0.18 to 0)

log⁡(*s*)	0.848 ± 0.100 (range, 0.697 to 1.030)

OSI	0.899 ± 0.369 (range, 0.422 to 1.589)

D = diopter, logMAR = logarithm of the minimal angle of resolution, log⁡(*s*) = log(straylight), and OSI = objective scattering index.

**Table 2:** Results from the Bland and Altman analysis for the first session (intrasession repeatability).

Parameters	Between first and second measurements		Between first and third measurements		Between second and third measurements	
	Mean difference	95% LoA	Mean difference	95% LoA	Mean difference	95% LoA
log⁡(*s*)	−0.002	−0.211 to 0.207	0.003	−0.240 to 0.245	0.005	−0.166 to 0.176
OSI	0.088	−0.302 to 0.477	0.018	−0.513 to 0.548	−0.070	−0.718 to 0.578

LoA = limit of agreement, log⁡(*s*) = log(straylight), and OSI = objective scattering index.

**Table 3:** Results from the Bland and Altman analysis between sessions (intersession repeatability).

Parameters	Between first and second sessions		Between first and third sessions		Between second and third sessions	
	Mean difference	95% LoA	Mean difference	95% LoA	Mean difference	95% LoA
log⁡(*s*)	−0.015	−0.267 to 0.237	−0.002	−0.208 to 0.205	0.014	−0.138 to 0.165
OSI	0.012	−0.404 to 0.428	−0.025	−0.657 to 0.607	−0.037	−0.399 to 0.325

LoA = limit of agreement, log⁡(*s*) = log(straylight), and OSI = objective scattering index.
